# The association of triglyceride-glucose index and combined obesity indicators with chest pain and risk of cardiovascular disease in American population with pre-diabetes or diabetes

**DOI:** 10.3389/fendo.2024.1471535

**Published:** 2024-09-06

**Authors:** Dongze Zheng, Jiamiao Cai, Sifan Xu, Shiyan Jiang, Chenlin Li, Bin Wang

**Affiliations:** ^1^ Department of Cardiology, The First Affiliated Hospital of Shantou University Medical College, Shantou, China; ^2^ Human Phenome Institute, Shantou University Medical College, Shantou, Guangdong, China; ^3^ Department of Nephrology, Jieyang People’s Hospital, Jieyang, Guangdong, China; ^4^ Department of Cardiology, Jieyang People’s Hospital, Jieyang, Guangdong, China

**Keywords:** triglyceride-glucose index, obesity, pre-diabetes, diabetes, chest pain, cardiovascular disease

## Abstract

**Aim:**

To investigate the correlation of the triglyceride-glucose (TyG) index and its combined obesity indicators with chest pain and cardiovascular disease (CVD) in the pre-diabetes and diabetes population.

**Methods:**

This cross-sectional investigation encompassed 6488 participants with diabetes and pre-diabetes who participated in the National Health and Nutrition Examination Survey (NHANES) between 2007 and 2016. The association of the TyG and combined obesity index with chest pain and CVD was investigated using weighted logistic regression models and restricted cubic spline (RCS) analysis. The receiver operating characteristic (ROC) curve analysis was performed to compare different indicators.

**Results:**

In multivariate logistic regression fully adjusted for confounding variables, our analyses revealed significant associations between TyG, TyG-BMI, TyG-WC, and TyG-WHtR and chest pain, with adjusted ORs (95% CI) of 1.21 (1.05, 1.39), 1.06 (1.01, 1.11), 1.08 (1.04, 1.14), and 1.27 (1.08, 1.48), respectively. For total-CVD, the adjusted ORs (95% CI) were 1.32 (1.08, 1.61), 1.10 (1.03, 1.17), 1.13 (1.06, 1.19), and 1.63 (1.35, 1.97), respectively, among which TyG, TyG-WC, and TyG-WHtR present curvilinear associations in RCS analysis (all *P*-nonlinear < 0.05). Furthermore, the ROC curve showed that TyG-WC had the most robust predictive efficacy for total-CVD, coronary heart disease (CHD), and myocardial infarction (MI), while TyG-WHtR had the best predictive ability for angina and heart failure.

**Conclusion:**

There are significant associations of TyG and its related indicators with chest pain and total-CVD among the pathoglycemia population. TyG-WC and TyG-WHtR demonstrated superior predictive capability for the incidence of cardiovascular events.

## 1 Introduction

The prevalence of cardiovascular diseases (CVD) has been steadily increasing in recent years, emerging as a significant global public health concern. According to data from the NHANES dataset spanning 2017 to March 2020, the overall prevalence of CVD was approximately 48.6%, affecting an estimated 127.9 million individuals in 2020. In the American Heart Association’s 2023 update on heart disease and stroke statistics, CVD was attributed to approximately 19.05 million deaths globally in 2020 (1–3). Additionally, chest pain, a marker of cardiovascular risk, has been understudied for an extended period. In the United States, the cumulative incidence of chest pain ranges from 20% to 40% (4). Compared to pain-free populations, patients in the general population who report chest pain have a higher future incidence of fatal and non-fatal CVD (5). Therefore, it is crucial to discover dependable indicators that can forecast and detect individuals with a high risk of chest pain and CVD at an early stage. Additionally, it is essential to promptly implement interventions to address risk factors in order to decrease the occurrence of cardiovascular events and alleviate the imminent danger to human life.

Recently, insulin resistance (IR), which is the dysfunction to utilize insulin of tissue cells and always accompany hyperinsulinemia, has been proven to be associated with lots of metabolic diseases, including CVD, dyslipidemia, obesity, and type 2 diabetes (6–9). However, the gold standard of IR is complex and expensive, which limited its application clinically (10). The triglyceride-glucose (TyG) index, which was a parameter calculated from the fasting triglyceride and fasting blood glucose levels, has been recently regarded as one of the biomarkers for IR (11–13). Growing studies have shown that TyG was associated with the prevalence of cardiovascular events such as coronary heart disease (CHD), heart failure (HF), myocardial infarction (MI), and so on (14–18). However, current research focuses on the whole population, and the participants specifically focused on dysglycemia were scattered. Obesity is a prevalent metabolic disorder that is globally recognized as a significant health concern, which is often accompanied by hypertension and abnormal lipid metabolism. Central obesity serves as a crucial indicator for evaluating the risk of various chronic disorders associated with obesity (19, 20). The indicators of central obesity included body mass index (BMI), waist circumference (WC) and waist-to-height ratio (WHtR). Recently, the combination of TyG with BMI, WC, and WHtR has been proposed as a novel indicator for IR. This combination has been found to be linked to many metabolic disorders, such as diabetes, metabolic syndrome, and non-alcoholic fatty liver disease (21–23).

Diabetes mellitus is widely recognized as a risk factor for CVD, and as high as 32% of patients with type 2 diabetes mellitus have cardiovascular disease (24). Recent epidemiological research has demonstrated a strong association between these TyG-related indicators and ASCVD (25, 26). In fact, several of these parameters have shown superior predictive ability compared to TyG. However, the link between TyG-related indicators and CVD in patients with dysglycemia has not been thoroughly investigated. Therefore, our research aimed to explore the potential correlation between TyG and its combined obesity indicators and chest pain and CVD within the diabetes and pre-diabetes population.

## 2 Method

### 2.1 Study design

The National Health and Nutrition Examination Survey (NHANES) is a nationwide representative survey that has been conducted in the United States since 1999 and continues to the present. In order to assess nutritional and physiological status, complex, multistage, stratified sampling is implemented from a nationally representative, non-institutionalized US civilian population. The study was conducted in accordance with the principles of the Declaration of Helsinki. The National Center for Health Statistics Research Ethics Review Board granted its approval. All individuals signed written informed consent.

### 2.2 Study population

**Figure 1:**
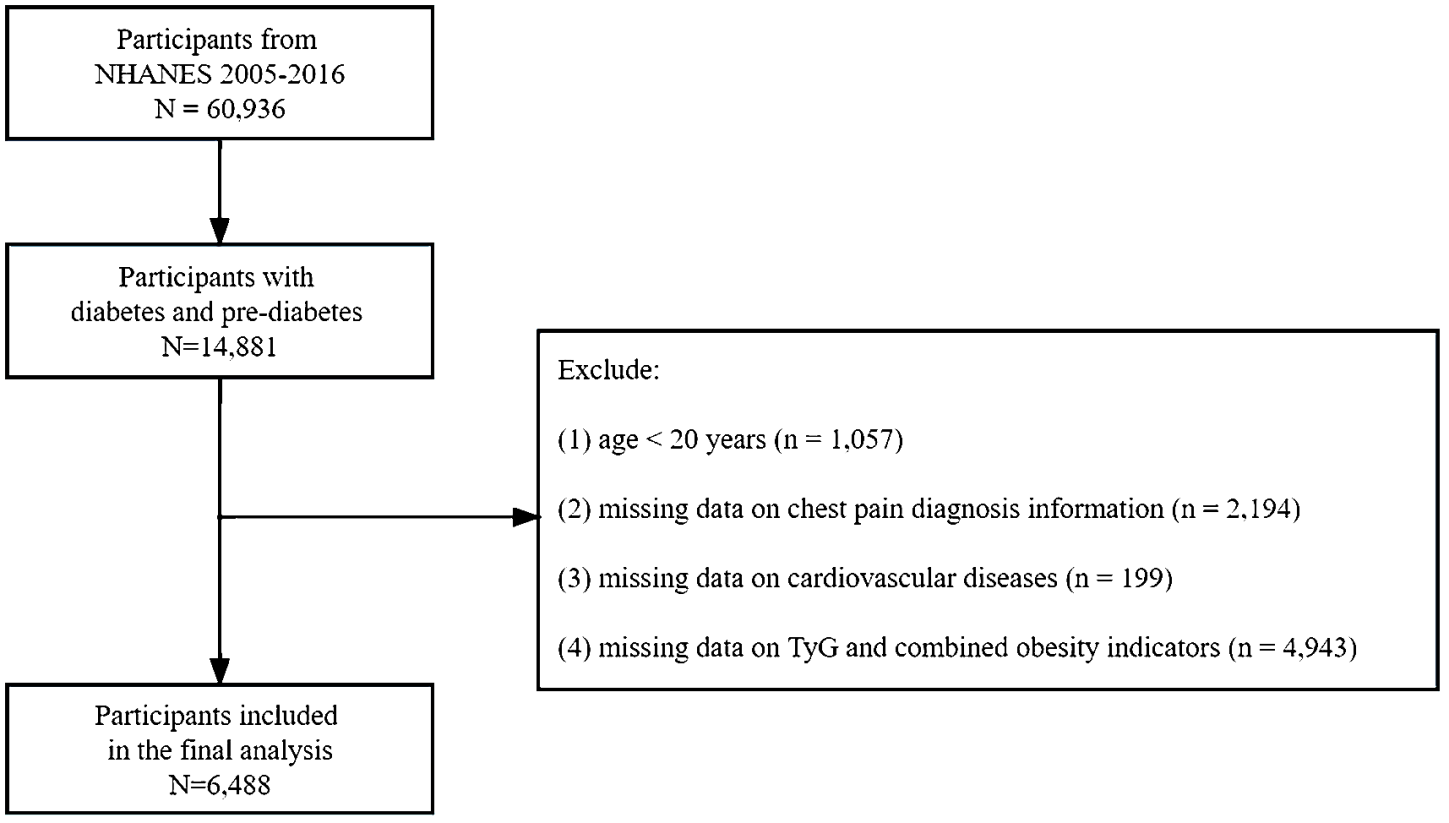
Flow chart of the study participants.

The data collected included structured interviews conducted in participants’ homes, health examinations conducted in mobile examination centers, and laboratory analyses of specimens. The study data can be accessed at https://www.cdc.gov/nchs/nhanes. Diabetes is defined as a self-reported diagnosis, the use of insulin or glycemic medication, hemoglobin A1c (HbA1c) ≥ 6.5%, fasting plasma glucose (FPG) ≥ 126 mg/dL, or 2-h blood glucose ≥ 200 mg/dL. Pre-diabetes is indicated by FPG levels between 100 mg/dL and 125 mg/dL, 2-h blood glucose between 140 mg/dL and 199 mg/dL, or HbA1c levels between 5.7% and 6.4% (27). This study included patients diagnosed with diabetes or prediabetes between 2007 and 2016. The following patients were excluded from the study (1): Participants under the age of 20 (2). Participants without data on chest pain diagnosis and cardiovascular diseases (3). Participants with missing baseline records on fasting blood triglyceride, fasting blood glucose, body weight, body height, or WC. Ultimately, 6,488 individuals with diabetes or pre-diabetes were enrolled following the screening process (**Figure 1**).

### 2.3 Calculation of TyG index and related parameters

Fasting blood triglycerides were measured using three different analyzers (Roche Hitachi 717/912, Roche modular P chemistry, and Roche/Hitachi Cobas 6000). Fasting blood glucose (FBG) measurement utilized two instruments (Roche C501 from 2007 to 2015 and Roche C311 in 2016). Considering different instruments for these indicators was not necessary under NHANES analysis guidelines. Body weight, height, WC and blood pressure were collected by trained health technicians when participants attended the health examinations. The health technician was assisted by a recorder during the body measurement examination. TyG index = ln [TG (mg/dL) × FBG (mg/dL)/2], TyG-WC index = TyG × WC (cm), TyG-BMI index = TyG × BMI (kg/m^2^), TyG-WHtR index = TyG × WC (cm)/height (cm) (28).

### 2.4 Outcome variables

Chest pain status and CVD were the primary outcomes. Chest pain was diagnosed when the participant responded “yes” to the question “{Have you/Has SP} ever had any pain or discomfort in {your/her/his} chest?” The diagnosis of CVD is determined by trained assessors, who obtain self-reported medical diagnoses during individual interview. Participants were inquired “ Has a doctor or other health professional ever told you that you have congestive heart failure, coronary heart disease, angina, myocardial infarction, or stroke?” Individuals who responded “yes” to any of these inquiries were diagnosed with CVD.

### 2.5 Assessment of covariates

We incorporated a diverse array of covariates that could have potentially impacted the results. All the participants provided information on age, sex, race (Mexican American, non-Hispanic white, non-Hispanic black, and other races), educational level (below high school, high school graduate or GED, and above high school), marital status (married/cohabiting, widowed/divorced/separated, and unmarried), poverty-income ratio (PIR), tobacco exposure (never, previous, and current), alcohol use (29) (non-drinking, moderate, heavy, or binge drinking), physical activity (30) (inactive, less active, active), chest pain status, and CVD through the survey questionnaires. We obtained SBP, DBP, and BMI through a physical examination. Laboratory results included LDL, TC, TBIL, SUA, and eGFR. The eGFR was calculated using the Chronic Kidney Disease Epidemiology Collaboration (CKD-EPI) equation (31).

### 2.6 Statistical analyses

This study used recommended NHANES weights to account for the disparities in sampling probabilities and nonresponse. Since data from five cycles from 2007 to 2016 were included in this study, the new weight index was constructed to be one-fifth of the original weight. Detailed information about the calculating weights can be found in https://wwwn.cdc.gov/nchs/nhanes/tutorials/Weighting.asp. Missing covariate values in survey data were addressed using a multilevel imputation approach (32). A Gibbs sampling procedure was used to generate 5 imputed data sets after a burn-in of 500 iterations and 100 updates to ensure stochastic independence between imputed data sets. Baseline characteristics were grouped based on the presence or absence of chest pain. Continuous variables are presented as mean (interquartile range, IQR) and were compared using the Wilcoxon rank-sum test. Categorical variables are expressed as frequency (percentages) and were compared by the Chi-square tests. The primary outcomes (chest pain and CVD) were examined in relation to TyG and its derivatives using multivariable logistic regression models. Additionally, in order to investigate the correlation between the occurrence of primary outcomes and TyG and its derivatives, smooth curve-fitting logistic regression models and restricted cubic splines (RCS) were implemented for exploratory analysis. In instances where the relationship was determined to be non-linear, logistic regression models were conducted separately on either side of the inflection point to investigate the correlation between TyG and its derivatives and the occurrence of various outcomes. Finally, receiver operating characteristic (ROC) curves were plotted to assess their efficacy in the prediction of CVD. We also conducted an analysis of five CVD. A two-tailed *P* value of < 0.05 was established as the threshold for statistical significance. All the statistical analyses were performed using R software version 4.3.1.

## 3 Results

### 3.1 Baseline characteristics

This study enrolled 6488 participants, of whom 1728 (26%) experienced chest pain at least once (**Table 1**). Those participants with chest pain tended to be male, older, white, people, smoke more, drink more, had higher educational statements, and had lower PIR. Furthermore, a higher proportion of participants with chest pain also reported a history of CVD, CHD, MI, HF, and stroke. In addition, participants with chest pain were more likely to have higher blood pressure and a higher BMI. TyG, TyG-BMI, TyG-WC, and TyG-WHtR were significantly higher in the chest pain group.

**Table 1:** Baseline characteristics of study participants.

Characteristic	Overall, N = 6,488(100%)	No chest pain, N = 4,760 (74%)	Chest pain, N = 1,728 (26%)	P Value
Age (years)	59.0 (50.0, 68.0)	58.0 (50.0, 68.0)	59.0 (49.0, 68.0)	0.9
Gender				**0.04**
Male	3,386 (52%)	2,493 (51%)	893 (54%)	
Female	3,102 (48%)	2,267 (49%)	835 (46%)	
Race				**0.008**
Mexican American	1,003 (6.8%)	762 (7.1%)	241 (6.0%)	
Non-Hispanic White	2,882 (72%)	2,045 (71%)	837 (73%)	
Non-Hispanic Black	1,328 (10%)	958 (9.8%)	370 (11%)	
Other Race	1,275 (11%)	995 (12%)	280 (9.9%)	
Education				0.1
Below high school	2,017 (20%)	1,461 (19%)	556 (22%)	
High school graduate or GED	1,514 (24%)	1,114 (24%)	400 (25%)	
High school or above	2,957 (55%)	2,185 (56%)	772 (53%)	
Marriage				0.5
Married or cohabiting	4,088 (68%)	3,046 (69%)	1,042 (67%)	
Widowed, divorced or separated	1,895 (25%)	1,357 (24%)	538 (26%)	
Unmarried	505 (7.1%)	357 (7.1%)	148 (7.0%)	
PIR	3.06 (1.62, 5.00)	3.20 (1.69, 5.00)	2.68 (1.31, 4.67)	**<0.001**
Smoking				**<0.001**
Never smoking	3,400 (51%)	2,597 (54%)	803 (44%)	
Previous smoking	2,232 (36%)	1,596 (34%)	636 (39%)	
Currently smoking	856 (13%)	567 (12%)	289 (17%)	
Drinking				**0.046**
Non drinking	1,924 (23%)	1,450 (24%)	474 (22%)	
Moderate drinking	2,629 (45%)	1,906 (45%)	723 (47%)	
Heavy drinking	1,298 (22%)	959 (23%)	339 (21%)	
Binge drinking	637 (9.0%)	445 (8.4%)	192 (11%)	
Exercise				0.4
Inactive	2,010 (27%)	1,466 (27%)	544 (27%)	
Less active	1,216 (19%)	863 (19%)	353 (21%)	
Active	3,262 (54%)	2,431 (54%)	831 (52%)	
Hypertension	4,394 (65%)	3,135 (62%)	1,259 (71%)	**<0.001**
Total-CVD	1,139 (16%)	533 (9.8%)	606 (32%)	**<0.001**
Coronary heart disease	458 (6.8%)	185 (3.8%)	273 (15%)	**<0.001**
Angina pectoris	281 (4.1%)	66 (1.3%)	215 (12%)	**<0.001**
Myocardial infarction	461 (6.4%)	161 (2.9%)	300 (16%)	**<0.001**
Congestive heart failure	344 (4.6%)	144 (2.6%)	200 (10%)	**<0.001**
Stroke	364 (4.8%)	215 (3.6%)	149 (8.2%)	**<0.001**
BMI	29 (26, 34)	29 (26, 34)	30 (26, 35)	**0.009**
SBP(mmHg)	125 (115, 138)	125 (115, 138)	124 (115, 137)	0.052
DBP(mmHg)	73 (65, 85)	74 (65, 87)	73 (64, 84)	**0.021**
Triglyceride(mmol/L)	1.37 (0.96, 2.00)	1.34 (0.95, 1.95)	1.45 (0.99, 2.14)	**0.006**
HDL(mmol/L)	1.29 (1.09, 1.58)	1.29 (1.09, 1.60)	1.24 (1.03, 1.53)	**<0.001**
LDL(mmol/L)	2.95 (2.33, 3.62)	2.97 (2.35, 3.65)	2.92 (2.25, 3.60)	**0.048**
TC(mmol/L)	5.04 (4.27, 5.79)	5.07 (4.32, 5.79)	4.91 (4.16, 5.74)	**0.013**
TBIL(umol/L)	12.0 (8.6, 15.4)	12.0 (8.6, 15.4)	12.0 (8.6, 15.4)	**0.038**
FBG(mmol/L)	6.05 (5.72, 6.72)	6.05 (5.66, 6.72)	6.00 (5.72, 6.67)	0.5
2h-OGTT(mmol/L)	7.33 (5.83, 9.27)	7.38 (5.94, 9.33)	7.11 (5.61, 9.10)	**0.012**
FBI(pmol/L)	12 (7, 18)	12 (7, 18)	12 (8, 19)	**0.033**
HbA1c(%)	5.80 (5.50, 6.10)	5.80 (5.40, 6.10)	5.80 (5.50, 6.10)	0.4
SUA(umol/L)	339 (286, 393)	339 (286, 393)	345 (286, 405)	**0.043**
SCR(umol/L)	78 (65, 90)	78 (65, 90)	80 (66, 91)	**0.042**
eGFR(ml/min/1.73m2)	90 (75, 102)	90 (75, 102)	90 (74, 102)	0.5
TyG	8.84 (8.45, 9.25)	8.82 (8.44, 9.23)	8.89 (8.48, 9.30)	**0.006**
TyG-BMI	262 (224, 308)	259 (223, 304)	268 (229, 315)	**0.002**
TyG-WC	919 (812, 1,038)	907 (806, 1,028)	952 (836, 1,062)	**<0.001**
TyG-WHtR	5.46 (4.84, 6.17)	5.41 (4.82, 6.12)	5.61 (4.93, 6.29)	**<0.001**

GED, general educational development test; PIR, poverty-income ratio; CVD, cardiovascular disease; BMI, body mass index; SBP, systolic blood pressure; DBP, diastolic blood pressure; HDL, high-density lipoprotein; LDL, low-density lipoprotein; TC, total cholesterol; TBIL, total bilirubin; FBG, fasting blood glucose; OGTT, oral glucose tolerance test; FBI, fasting blood insulin; HbA1c, hemoglobin A1c; SUA, serum uric acid; SCR, serum creatinine; eGFR, estimated glomerular filtration rate; TyG, Triglyceride-glucose index; TyG-WC, Triglyceride-glucose-waist circumference index; TyG-WHtR, Triglyceride-glucose-waist-to-height radio index.

Bold values indicate p value of < 0.05.

### 3.2 Association between TyG and related parameters and outcomes

**Figure 2:**
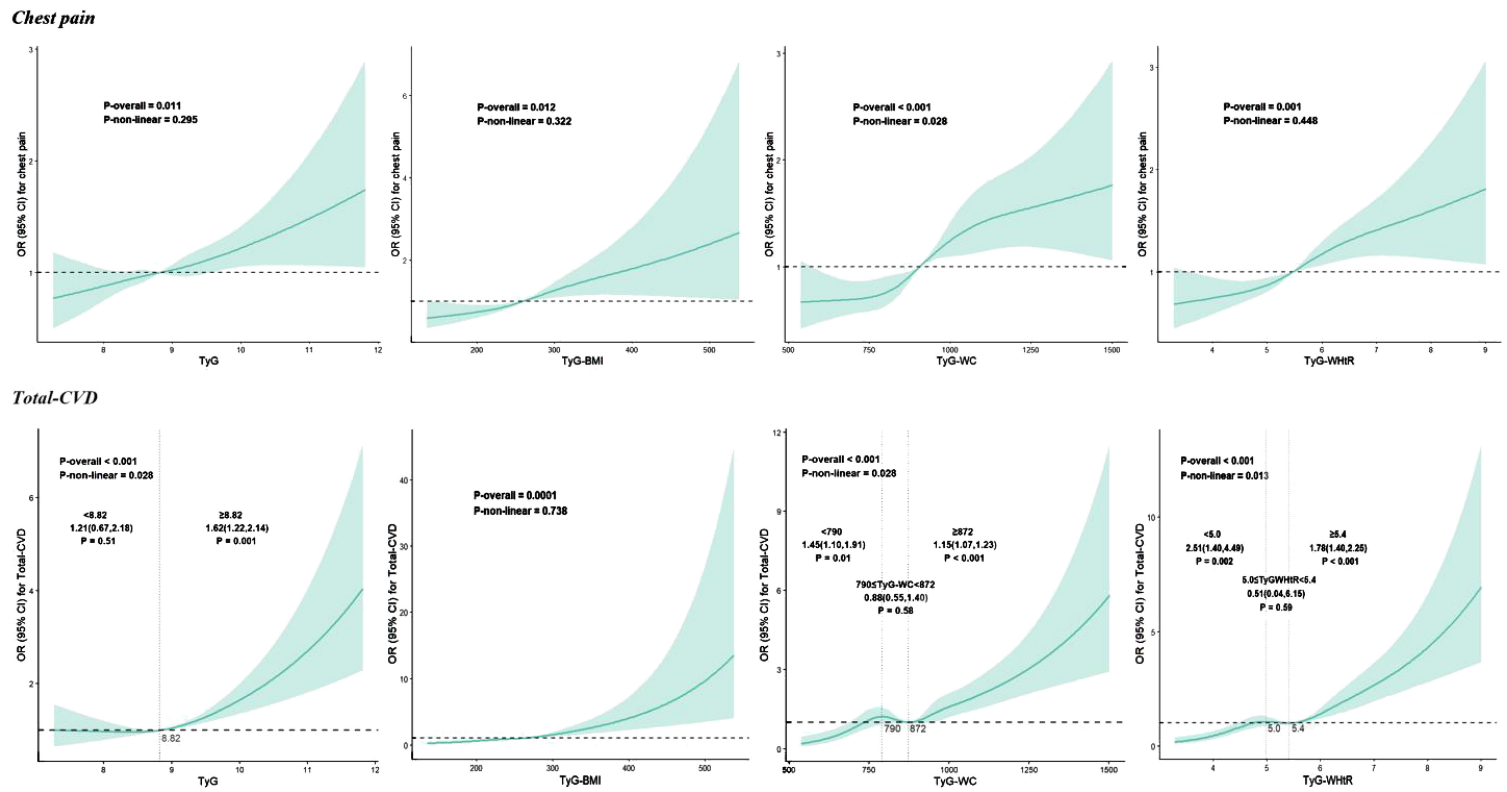
Restricted cubic spline curves for TyG, TyG-BMI, TyG-WC and TyG-WHtR associated with chest pain and total-CVD. CVD, cardiovascular diseases; TyG, Triglyceride-glucose index; TyG-WC, Triglyceride-glucose-waist circumference index; TyG-WHtR, Triglyceride-glucose-waist-to-height radio index.

**Table 2** shows the association between the study variable and chest pain. After adjusting for covariates, TyG (OR 1.21, 95% CI: 1.05-1.39, *P* = 0.01), TyG-BMI (OR 1.06, 95% CI: 1.01-1.11, *P* = 0.02), TyG-WC (OR 1.09, 95% CI: 1.04-1.14, *P* < 0.001) and TyG-WHtR (OR 1.27, 95% CI: 1.08-1.48, *P* = 0.004) as continuous variables were significantly and positively associated with chest pain. When analyzing these parameters as categorical variates, there was no statistically significant relationship between TyG and chest pain, while the combined predictors were all proven to increase the risk of chest pain in model 3. For participants in the fourth quartile of TyG-BMI, TyG-WC, and TyG-WHtR, the risk of chest pain increased by respectively 65%, 71%, and 70% (TyG-BMI: OR 1.65, 95% CI: 1.07-2.55, *P* = 0.02; TyG-WC: OR 1.71, 95% CI: 1.19-2.46), *P* = 0.004; TyG-WHtR: OR 1.70, 95% CI: 1.19-2.44, *P* = 0.004). The RCS analysis presents the association between TyG, TyG-BMI, TyG-WC, and TyG-WHtR and chest pain more visually. Significant linear correlations were found between four parameters and chest pain (all *P*-nonlinear > 0.05) (**Figure 2**).

**Table 2:** ORs (95% CIs) for chest pain according to TyG, TyG-BMI, TyG-WC and TyG-WHtR.

	Model 1 OR (95% Cl)	*P* value	Model 2 OR (95% Cl)	*P* value	Model 3 OR (95% Cl)	*P* value
**TyG (continuous)**	1.22 (1.09,1.37)	**<0.001**	1.23 (1.10,1.38)	**<0.001**	1.21 (1.05,1.39)	**0.01**
TyG (categorical)						
Q1	Reference		Reference		Reference	
Q2	0.98 (0.79,1.21)	0.82	0.99 (0.79,1.22)	0.9	0.93 (0.73,1.17)	0.53
Q3	1.18 (0.93,1.50)	0.17	1.21 (0.95,1.54)	0.13	1.19 (0.90,1.56)	0.22
Q4	1.30 (1.02,1.64)	**0.03**	1.32 (1.04,1.68)	**0.02**	1.26 (0.96,1.66)	0.1
**TyG-BMI** **(continuous, per 10 increase)**	1.02 (1.01,1.03)	**0.003**	1.02 (1.01,1.03)	**0.003**	1.06 (1.01,1.11)	**0.02**
TyG-BMI (categorical)						
Q1	Reference		Reference		Reference	
Q2	1.08 (0.84,1.38)	0.55	1.07 (0.83,1.36)	0.6	1.18 (0.91,1.52)	0.2
Q3	1.35 (1.12,1.64)	**0.003**	1.36 (1.12,1.65)	**0.003**	1.43 (1.08,1.89)	**0.01**
Q4	1.42 (1.13,1.78)	**0.003**	1.42 (1.12,1.80)	**0.004**	1.65 (1.07,2.55)	**0.02**
**TyG-WC** **(continuous, per 50 increase)**	1.06 (1.03,1.08)	**<0.0001**	1.05 (1.03,1.08)	**<0.0001**	1.09 (1.04,1.14)	**<0.001**
TyG-WC (categorical)						
Q1	Reference		Reference		Reference	
Q2	1.00 (0.84,1.19)	0.97	0.99 (0.83,1.18)	0.91	1.06 (0.87,1.29)	0.57
Q3	1.44 (1.17,1.78)	**<0.001**	1.42 (1.15,1.76)	**0.001**	1.54 (1.17,2.02)	**0.003**
Q4	1.58 (1.29,1.93)	**<0.0001**	1.55 (1.26,1.90)	**<0.0001**	1.71 (1.19,2.46)	**0.004**
**TyG-WHtR (continuous)**	1.17 (1.08,1.26)	**<0.001**	1.18 (1.09,1.27)	**<0.0001**	1.27 (1.08,1.48)	**0.004**
TyG-WHtR (categorical)						
Q1	Reference		Reference		Reference	
Q2	1.06 (0.88,1.28)	0.54	1.07 (0.88,1.29)	0.5	1.16 (0.94,1.43)	0.16
Q3	1.33 (1.06,1.66)	**0.01**	1.35 (1.08,1.69)	**0.01**	1.40 (1.05,1.86)	**0.02**
Q4	1.52 (1.24,1.87)	**<0.0001**	1.56 (1.27,1.92)	**<0.0001**	1.70 (1.19,2.44)	**0.004**

Model 1was unadjusted, Model 2 was adjusted for age, gender and race, Model 3 was adjusted for age, gender, race, smoking, drinking, exercise, education, SBP, DBP, BMI, LDL, TC, TBIL, SUA, eGFR, coronary heart disease, angina, angina pectoris, myocardial infarction, congestive heart failure, and stroke. TyG, Triglyceride-glucose index; TyG-WC, Triglyceride-glucose-waist circumference index; TyG-WHtR, Triglyceride-glucose-waist-to-height radio index.

Bold values indicate p value of < 0.05.

**Table 3** illustrates the relationship between TyG and related parameters and total-CVD. TyG (OR 1.32, 95% CI: 1.08-1.61, *P* = 0.01), TyG-BMI (OR 1.10, 95% CI: 1.03-1.17, *P* = 0.01), TyG-WC (OR 1.13, 95% CI: 1.06-1.19, *P* < 0.0001), and TyG-WHtR (OR 1.63, 95% CI: 1.35-1.97, *P* < 0.0001) were all significantly and positively associated with total-CVD after adjusting for covariates. When these parameters were analyzed as categorical variates, TyG, TyG-WC, and TyG-WHtR were all demonstrated to increase the risk of total-CVD, whereas there was no statistically significant relationship between TyG-BMI and total-CVD in model 3. The risk of chest pain increased by 42%, 76%, and 109% for participants in the fourth quartile of TyG, TyG-WC, and TyG-WHtR, respectively (TyG: OR 1.65, 95% CI: 1.07-2.55, *P* = 0.04; TyG-WC: OR 1.71, 95% CI: 1.19-2.46), *P* = 0.01; TyG-WHtR: OR 1.70, 95% CI: 1.19-2.44, *P* = 0.001). As shown in **Figure 2**, only TyG-BMI was observed to have a linear relationship (*P*-nonlinear > 0.05) after adjustments. TyG, TyG-WC, and TyG-WHtR exhibited non-linear associations (all *P*-nonlinear <0.05) with total-CVD. Interestingly, a J-shaped relationship was observed between TyG and total total-CVD, while TyG-WC and TyG-WHtR show a temporary plateau type curve with total-CVD.

**Table 3:** ORs (95% CIs) for cardiovascular diseases according to TyG, TyG-BMI, TyG-WC and TyG-WHtR.

	Model 1 OR (95% Cl)	*P* value	Model 2 OR (95% Cl)	*P* value	Model 3 OR (95% Cl)	*P* value
**TyG(continuous)**	1.23 (1.09,1.40)	**0.002**	1.43 (1.23,1.66)	**<0.0001**	1.34 (1.11,1.62)	**0.003**
TyG(categorical)						
Q1	Reference		Reference		Reference	
Q2	1.10 (0.84,1.43)	0.48	1.15 (0.86,1.52)	0.34	1.07 (0.79,1.47)	0.65
Q3	1.14 (0.91,1.43)	0.25	1.24 (0.95,1.60)	0.11	1.04 (0.75,1.44)	0.82
Q4	1.38 (1.05,1.82)	**0.02**	1.73 (1.29,2.33)	**<0.001**	1.46 (1.05,2.04)	**0.02**
**TyG-BMI** **(continuous, per 10 increase)**	1.03 (1.01,1.04)	**<0.001**	1.06 (1.04,1.07)	**<0.0001**	1.10 (1.04,1.17)	**0.002**
TyG-BMI(categorical)						
Q1	Reference		Reference		Reference	
Q2	1.06 (0.80,1.39)	0.69	1.15 (0.87,1.51)	0.32	1.07 (0.76,1.49)	0.7
Q3	1.37 (1.07,1.76)	**0.01**	1.73 (1.33,2.26)	**<0.0001**	1.37 (0.96,1.95)	0.08
Q4	1.48 (1.17,1.89)	**0.002**	2.37 (1.81,3.11)	**<0.0001**	1.53 (0.88,2.67)	**0.13**
**TyG-WC** **(continuous, per 50 increase)**	1.09 (1.07,1.12)	**<0.0001**	1.13 (1.10,1.16)	**<0.0001**	1.13 (1.07,1.19)	**<0.0001**
TyG-WC(categorical)						
Q1	Reference		Reference		Reference	
Q2	1.20 (0.92,1.57)	0.18	1.14 (0.86,1.52)	0.35	1.03 (0.73,1.44)	0.88
Q3	1.52 (1.19,1.93)	**<0.001**	1.58 (1.22,2.04)	**<0.001**	1.32 (0.94,1.85)	0.11
Q4	2.12 (1.63,2.74)	**<0.0001**	2.62 (1.97,3.48)	**<0.0001**	1.81 (1.17,2.79)	**0.01**
**TyG-WHtR(continuous)**	1.38 (1.26,1.52)	**<0.0001**	1.53 (1.38,1.70)	**<0.0001**	1.65 (1.37,1.98)	**<0.0001**
TyG-WHtR(categorical)						
Q1	Reference		Reference		Reference	
Q2	1.08 (0.83,1.40)	0.59	1.06 (0.81,1.38)	0.67	1.02 (0.75,1.39)	0.92
Q3	1.46 (1.13,1.89)	**0.004**	1.51 (1.16,1.97)	**0.003**	1.33 (0.97,1.82)	0.08
Q4	2.23 (1.73,2.87)	**<0.0001**	2.74 (2.08,3.62)	**<0.0001**	2.14 (1.45,3.18)	**<0.001**

Model 1was unadjusted, Model 2 was adjusted for age, gender and race, Model 3 was adjusted for age, gender, race, smoking, drinking, exercise, marriage, education, PIR, SBP, DBP, BMI, LDL, TC, TBIL, SUA, eGFR, and chest pain. TyG, Triglyceride-glucose index; TyG-WC, Triglyceride-glucose-waist circumference index; TyG-WHtR, Triglyceride-glucose-waist-to-height radio index. Bold values indicate p value of < 0.05.

We also investigate CHD, angina, MI, HF, and stroke. When treated as a continuous variable, TyG (CHD: OR 1.49 95%CI 1.18-1.89, *P* = 0.001; HF: OR 1.38 95%CI 1.06-1.80, *P* = 0.02) and TyG-BMI (per 10 increases; CHD: OR 1.15 95%CI 1.07-1.24, *P* = 0.001; HF: OR 1.11 95%CI 1.02-1.20, *P* = 0.01) demonstrated a significantly positive correlation with CHD and HF, while having no statistically significant associations with angina and MI. Participants in the fourth quartile of TyG-WC experienced a 124%, 114%, and 81% increase in the likelihood of experiencing CHD, MI, and HF, respectively (CHD: OR 2.24 95%CI 1.22-4.11, *P* = 0.01; MI: OR 2.14 95%CI 1.20-3.83, *P* = 0.01; HF: OR 1.81 95%CI 1.13-2.91, *P* = 0.02). TyG-WHtR had the highest correlation with HF, with an OR of 2.72 in the fourth quartile (95% CI 1.46-5.09, *P* = 0.002). No significant relationships were found between the indicators and stroke. Further details are presented in **Supplementary Table 1**. In correlation with coronary disease, TyG demonstrated a J-shaped relationship. For HF, a U-shaped relationship was observed with TyG-WC. In the association between TyG-BMI, TyG-WHtR, and TyG and HF, linear relationships were observed (all *P*-nonlinear >0.05). Additionally, it showed linear associations between TyG-WC and MI (*P*-nonlinear > 0.05) and nonlinear associations between other indices and MI (all *P*-nonlinear < 0.05). **Supplementary Figures S1**-**S4** illustrated further details.

### 3.3 Threshold effect analysis

Additionally, for RCS curves that exhibited curvilinear relationships previously mentioned, segmented logistic regression models were utilized to investigate the specific effects before and after the inflection points. We discovered that the cardiovascular risk increased as the TyG index increased when it exceeded 8.82. In contrast, this trend was absent when the TyG index was less than 8.82. We also observed a consistent trend between the TyG index and the risk of coronary artery disease (<8.82: OR = 1.46, 95% CI 0.73–2.90, *P* = 0.28; >8.82: OR = 1.96, 95% CI 1.43–2.6, *P* < 0.001), as well as between the TyG-WC and the risk of HF (per 50 unit increase; 814: OR = 1.06, 95% CI 0.76–1.47, *P* = 0.75; >814: OR = 1.21, 95% CI 1.11–1.31, *P* < 0.001). The risk of total-CVD increases by 46% (OR = 1.46, 95% CI 1.11–1.92, *P* = 0.01) and 15% (OR = 1.15, 95% CI 1.07–1.24, *P* = 0.001) when the TyG-WC is less than 790 and higher than 872, respectively, for per 50 unit increase in TyG-WC, while there is a plateau between 790 and 872 (OR = 0.87, 95% CI 0.54–1.39, *P* = 0.55). When the TyG-WHtR<5.0 or ≥ 5.4, increased TyG-WHtR was significantly associated with increased risk of total-CVD, but there was no significant association when TyG-WHtR between 5.0 and 5.4. Similarly, TyG-WHtR also demonstrated this trend in MI, with inflection points at 4.7 and 5.2, respectively. More details are available in **Supplementary Tables S2**-**S5**.

### 3.4 Performance and predictive ability of TyG and related parameters

The ROC curve showed that TyG-WC exhibited the highest diagnostic accuracy for total-CVD (AUC: 0.591), followed by TyG-WHtR (AUC: 0.590). TyG-WC also demonstrated superior diagnostic efficiency for CHD and MI, with AUCs of 0.588 and 0.581, respectively. TyG-WHtR followed with AUCs of 0.568 for CHD and 0.564 for MI, outperforming TyG-BMI and TyG in both cases. For HF, TyG-WHtR had the highest diagnostic efficacy (AUC 0.614), while TyG-WC was slightly inferior to TyG-WHtR (AUC 0.610). Moreover, TyG demonstrated superior diagnostic efficacy for angina (AUC 0.599) (**Table 4**; **Supplementary Figure S5**).

**Table 4:** Results of ROC analysis.

	AUC (95% CI)	Cutoff Value	Sensitivity (%)	Specificity (%)	Youden Index
Total-CVD					
TyG	0.535 (0.517-0.554)	9.107	37.1	69.0	0.061
TyG-BMI	0.551 (0.533-0.569)	268.8	50.1	57.9	0.080
TyG-WC	0.591 (0.574-0.609)	904.9	62.2	52.0	0.142
TyG-WHtR	0.590 (0.572-0.608)	5.494	60.1	53.3	0.135
Coronary heart disease					
TyG	0.549 (0.521-0.577)	9.333	29.0	79.4	0.084
TyG-BMI	0.530 (0.503-0.556)	251.8	62.0	45.2	0.072
TyG-WC	0.588 (0.562-0.614)	891.9	67.2	46.5	0.137
TyG-WHtR	0.568 (0.542-0.594)	5.407	63.3	48.2	0.116
Angina pectoris					
TyG-WHtR	0.599 (0.566-0.631)	5.486	67.6	51.5	0.191
Myocardial infarction					
TyG-WC	0.581 (0.554-0.608)	953.8	52.3	61.8	0.141
TyG-WHtR	0.564 (0.538-0.591)	5.449	61.8	49.9	0.117
Congestive heart failure					
TyG	0.544 (0.512-0.576)	9.323	29.1	78.7	0.078
TyG-BMI	0.575 (0.543-0.607)	267.9	55.8	56.5	0.123
TyG-WC	0.610 (0.578-0.642)	942.0	60.2	59.8	0.200
TyG-WHtR	0.614 (0.582-0.645)	5.584	62.5	55.6	0.181

AUC, Area under curve; CI, Confidence Interval; CVD, cardiovascular diseases; TyG, Triglyceride-glucose index; TyG-WC, Triglyceride-glucose-waist circumference index; TyG-WHtR, Triglyceride-glucose-waist-to-height radio index.

## 4 Discussion

To our knowledge, this study represents the first investigation into the associations of TyG and combined obesity indices with both chest pain and CVD among individuals with diabetes and pre-diabetes. Our study revealed that TyG and combined obesity indices were significantly associated with chest pain and total-CVD, among which TyG, TyG-WC, and TyG-WHtR present curvilinear associations for total-CVD. Furthermore, different associations of TyG and its related parameters with the occurrence of different CVD in individuals with dysglycemia were observed. Among the above indicators, TyG-WC had the most robust predictive efficacy for total-CVD, CHD, and MI, while TyG-WHtR had the best predictive ability for angina pectoris and HF.

The TyG index, a measure of insulin sensitivity (33, 34), has emerged as a significant risk factor for various metabolic disorders, including diabetes, pre-diabetes, obesity, CVD and renal insufficiency (35–39). Recent studies have suggested that a higher TyG index was associated with an increased risk of varying cardiovascular events, including CHD, MI, carotid plaque, and so on (40–42). A meta-analysis further underscored that higher TyG was significantly associated with an increased risk of ASCVD (43). Another retrospective cohort study demonstrated that higher TyG was associated with a poorer prognosis for MI patients with diabetes (44). However, conflicting findings emerged from another study. Schmiegelow et al. found that IR was associated with CVD risk after adjustment for age and race, but this association was no longer significant after adjustment for HDL-C. There are two potential explanations for this. On the one hand, the study was limited to postmenopausal women and may not be generalizable to younger women or men. On the other hand, strokes that could not be classified as ischemic or hemorrhagic strokes were excluded, and some data may have been missed. In addition, the relationship between TyG and HF was also confirmed previously (45, 46). Our study indicated that TyG may serve as a predictive marker for total-CVD, CHD, MI, and HF, consistent with prior research findings. Yao et al. demonstrated a positive linear relationship between TyG and chest pain (47). Similarly, the correlation between TyG and chest pain was confirmed among the pathoglycemia population after adjustment in Model 3.

Obesity is well-established as a risk factor for CVD, and previous research has also linked obesity to IR and diabetes (48, 49). Therefore, TyG combined obesity indicators were brought into study in the recent year. Notably, our study focused on the correlation between TyG combined with obesity indicators and cardiovascular events in the pathoglycemia population. Overall, the result demonstrated that individuals with higher TyG-BMI, TyG-WC, and TyG-WHtR were more likely to have an increased risk of total-CVD, CHD, MI, and HF. In our research, TyG-WC and TyG-WHtR showed the best correlation with CHD and HF, while TyG-BMI had the best correlation with CHD. Dang et al. revealed that TyG-WC, TyG-WHtR, and TyG-BMI were positively associated with cardiovascular events mentioned above, and both TyG-WC and TyG-WHtR had more robust predictive efficacy than TyG in the American population, which was in line with our research (50). ROC analysis showed that TyG-WC may be a better predictor for total-CVD, MI, and coronary disease than other indicators, while TyG-WHtR may be better at predicting HF than other indicators. Indeed, most of the current studies have focused on the effects of the current most popular TyG, while studies on these TyG-related parameters are still relatively scattered. Prior studies demonstrated the superiority of the TyG-related index as a risk factor for metabolic diseases, which are always cooccurrences with cardiovascular events (51). Another cross-sectional study conducted in Tawan suggested that higher TyG, TyG-BMI, and TyG-WC were accompanied by a higher risk of CVD as well (52). In accordance, a 15-year follow-up prospective cohort study enrolled 98191 participants and demonstrated that TyG-WC and TyG-WHtR showed better capacity in predicting ASCVD compared with TyG (AUC: TyG: 0.583; TyG-WC: 0.612; TyG-WHtR: 0.613) (28). Nevertheless, it’s worth noting that some of the previous studies demonstrated contrasting results. A prospective cohort study confirmed significantly and positively correlation between TyG-related index and cardiovascular diseases (TyG-WC: HR: 1.256 95%CI 1.090-1.447; TyG-BMI: HR: 1.343 95%CI 1.183-1.525; TyG-WHtR: HR: 1.252 95%CI 1.088-1.441) (53). However, this study pointed out non-significant correlation of TyG-related indicators in diabetes population, which is quite distinct with us. Considering that pathoglycemia individuals generally have higher surrogate values of IR, the association with CVD is more complex relative to normal group. Moreover, a retrospective observational cohort study suggested TyG perform better in predicting individuals at risk of incident cardiovascular events than TyG-related indicators, including TyG-WC and TyG-WHtR (54). This study found a different conclusion, whose primary outcome was incident ASCVD, including MI and ischemic stroke, and the fact that the study population was composed of individuals from various ethnic backgrounds may have contributed to the discrepancies. The mechanisms underlying the different predictive performance have not been fully elucidated, and the combined effects of IR and excess abdominal fat on CVD risk are a possible explanation. Insulin sensitivity, represented by TyG, and excessive accumulation of visceral adiposity, indicated by central adiposity measures, are both strongly associated with chronic inflammation, endothelial dysfunction, and atherosclerosis, all of which increase CVD risk. Those controversy results inspire more research to further explore the association of surrogate IR indexes with cardiovascular events. Furthermore, the majority of previous research concentrated on the overall population, with very few studies focusing on the pathoglycemia population or other specific individuals. Notably, our study found no significant link of the TyG and its related index with stroke in pathoglycemia individuals. However, a cohort study among rural Chinese people found a significant correlation between the TyG and combined obesity index and stroke (55). Given the strong association between diabetes, pre-diabetes, and CVD, excellent predictors seem to be crucial for earlier diagnosis and better prognosis, so that more research to further explore the potential relationship between TyG and related index with cerebrovascular disease makes great sense, particularly among pathoglycemia groups. Overall, more research on those indexes is critical for predicting cardiovascular events.

The TyG index is a measurement of insulin sensitivity in humans. Recently, IR can be defined as an impaired response to insulin stimulation of tissue cells so that hyperinsulinemia takes place consequently. IR was associated with kinds of metabolic syndromes, including type 2 diabetes, obesity, and CVD (56, 57). Obesity has been recognized as one of the most crucial factors for cardiovascular events for a long time, including dyslipidemia, IR, type 2 diabetes, hypertension, and so on (58, 59). Recently, with the interaction between IR and obesity, combined indexes have emerged to enhance predictive capability and facilitate earlier diagnosis. Although the underlying mechanism between TyG with TyG-related index and cardiovascular events still remains unclear, several potential pathophysiological pathways have been discussed in previous studies. Physiological concentrations of insulin will contribute to vascular relaxation and promote the production of nitric oxide in endothelial cells for tissue artery recruitment. Insulin-resistant states may hinder these physical processes, and consequently, hyperinsulinemia may encourage vascular constriction, potentially leading to vascular stiffening and even hypertension (60, 61). According to a study, insulin plays a role in mediating Na+ channels, and consequently, hyperinsulinemia leads to Na+ channel dysfunction, which further remodels the vasculature (62). Moreover, previous studies have shown that the dysfunction of EnNaC contributes to cardiac fibrosis, which is likely a crucial factor in the remodeling of cardiac diastolic dysfunction (63, 64). In addition, IR can cause downregulation of SGK-1 expression, which promotes adipocyte and immune cell dysfunction, resulting in dyslipidemia and abnormal systemic inflammation, and finally accelerate atherosclerosis (65–67). Finally, IR can contribute to plate adhesion, activation, and aggregation, leading to cardiac dysfunction and myocardial injury (68, 69). Overall, our research demonstrated that the TyG combined obesity index had a higher correlation with chest pain and cardiovascular events. We also revealed their superiority in predicting total-CVD, MI, angina, and coronary diseases simultaneously, with WC and WHtR demonstrating the most robust potential diagnosis efficacy for cardiovascular events in individuals with diabetes and pre-diabetes. The superiority of these combined indicators may stem from the fact that individuals with pathoglycemia are always associated with a higher risk of abdominal obesity or dyslipidemia. Numerous related studies have emphasized the potential of these emerging indexes, despite some differences with prior studies. There are reasons to believe that it makes sense to apply those indices to the clinic for earlier diagnosis and more timely interventions among the pathoglycemia population.

The study adds to existing knowledge by providing new evidence on how TyG and combined TyG-obesity indicators are linked to chest pain and cardiovascular events in both diabetic and pre-diabetic people. Furthermore, the research data comes from NHANES, which guarantees a large and representative sample for the study. Nevertheless, there still remain some limitations. Firstly, the samples included in this database only come from the United States, which may limit the generalizability of the findings. Populations in different regions may have different risk factors and prevalence of disease because of differences in genetics, environment, lifestyle, and health care systems. Future studies should replicate these findings in different countries and ethnic groups to assess whether the associations of TyG and TyG combined obesity indicators with chest pain and cardiovascular events are generalizable. Secondly, despite adjusting for numerous confounding factors, there is inevitably the influence of unmeasured or residual confounders affecting the results. For example, dietary patterns, genetic factors, and psychosocial factors, among others, may all be associated with the primary outcome variables studied but may not have been fully considered in this study. Future studies should consider including these factors to more accurately estimate the impact of TyG-related metrics. Thirdly, even though the NHANES questionnaire adheres to strict quality control, the validity of the study may still be impacted by the limitations of self-reported measurement and potential misclassification of results. If participants tended to underestimate or not report their chest pain and CVD status, this may have led to studies underestimating the true prevalence of these conditions. This underestimation may attenuate the actual strength of the association of TyG related measures with chest pain and CVD. Future studies should use more objective measures to reduce bias validation and strengthen the findings of the current study. Fourthly, due to the inherent limitations of the NHANES database, it is difficult for us to establish a causal relationship between chest pain and the survey variable and to further study the etiology of chest pain. Future studies should employ prospective cohort studies or randomized controlled trials to better understand the temporal order and potential causal pathways between these variables. Finally, the study did not discuss the inter-group differences between diabetes and pre-diabetes.

## 5 Conclusion

In our research, TyG and combined obesity indices were significantly associated with chest pain and total-CVD among the pathoglycemia population. Specifically, TyG-WC had the most robust predictive efficacy for total-CVD, CHD and MI, while TyG-WHtR had the best predictive ability for angina pectoris and HF. In the context of a growing disease burden and limited medical resources, this study identified predictive surrogate indexes for IR that are both convenient, cost-effective and well-predictive. It is expected to offer valuable references for the screening and prediction of potential cardiovascular disease risk in primary hospitals and communities with abnormal blood glucose.

## Data Availability

Publicly available datasets were analyzed in this study. This data can be found here: https://www.cdc.gov/nchs/nhanes/index.htm.
