# Supplementary material for: The association of triglyceride-glucose index and combined obesity indicators with chest pain and risk of cardiovascular disease in American population with pre-diabetes or diabetes
Source: Front Endocrinol (Lausanne). 2024 Sep 6;15:1471535. doi: 10.3389/fendo.2024.1471535 (PMC11412814; doi:10.3389/fendo.2024.1471535)
Supplement: Supplementary file 1 [file DataSheet1.docx]

Supplementary Material

**Supplementary Table 1** ORs (95% CIs) for coronary heart disease, angina, myocardial infarction, congestive heart failure, and stroke according to TyG, TyG‑BMI,TyG‑WC and TyG‑WHtR

|  | **Coronary heart disease** | ***P* value** | **Angina** | ***P* value** | **Myocardial infarction** | ***P* value** | **Congestive heart failure** | ***P* value** | **Stroke** | ***P* value** |
| --- | --- | --- | --- | --- | --- | --- | --- | --- | --- | --- |
| **TyG(continuous)** | 1.49(1.18,1.89) | **0.001** | 1.23(0.87, 1.76) | 0.24 | 1.09(0.83,1.42) | 0.53 | 1.38(1.06,1.80) | **0.02** | 0.95(0.70,1.30) | 0.76 |
| **TyG(categorical)** |  |  |  |  |  |  |  |  |  |  |
| Q1 | ref | ref | ref | ref | ref | ref | ref | ref | ref | ref |
| Q2 | 1.10(0.69,1.77) | 0.68 | 1.57(0.91, 2.71) | 0.11 | 1.11(0.74,1.66) | 0.62 | 1.02(0.65,1.60) | 0.92 | 0.89(0.60,1.32) | 0.55 |
| Q3 | 1.05(0.70,1.56) | 0.82 | 1.10(0.65, 1.86) | 0.72 | 0.88(0.57,1.38) | 0.59 | 1.07(0.61,1.85) | 0.82 | 0.72(0.45,1.15) | 0.16 |
| Q4 | 1.51(0.96,2.39) | 0.07 | 1.41(0.75, 2.66) | 0.28 | 1.28(0.80,2.04) | 0.29 | 1.51(0.96,2.37) | 0.07 | 0.94(0.57,1.55) | 0.8 |
| **TyG-BMI**  **(continuous, per 10 increase)** | 1.15(1.07,1.24) | **<0.001** | 1.07(0.96, 1.19) | 0.25 | 1.03(0.94,1.12) | 0.56 | 1.11(1.02,1.20) | **0.01** | 1.00(0.90,1.11) | 0.98 |
| **TyG-BMI(categorical)** |  |  |  |  |  |  |  |  |  |  |
| Q1 | ref | ref | ref | ref | ref | ref | ref | ref | ref | ref |
| Q2 | 1.30(0.84,2.02) | 0.24 | 0.93(0.50, 1.76) | 0.83 | 1.00(0.61,1.64) | 0.99 | 0.77(0.49,1.21) | 0.26 | 1.10(0.67,1.81) | 0.69 |
| Q3 | 2.01(1.21,3.35) | **0.01** | 1.75(0.89, 3.43) | 0.1 | 1.33(0.82,2.17) | 0.24 | 1.18(0.69,2.01) | 0.54 | 0.75(0.43,1.29) | 0.29 |
| Q4 | 2.34(1.16,4.74) | **0.02** | 1.98(0.78, 5.03) | 0.15 | 1.59(0.77,3.28) | 0.21 | 1.53(0.79,2.98) | 0.21 | 0.81(0.42,1.57) | 0.53 |
| **TyG-WC**  **(continuous, per 50 increase)** | 1.17(1.08,1.26) | **<0.0001** | 1.09(0.99, 1.20) | 0.09 | 1.08(1.00,1.16) | **0.04** | 1.15(1.07,1.24) | **<0.001** | 1.03(0.93,1.13) | 0.58 |
| **TyG-WC(categorical)** |  |  |  |  |  |  |  |  |  |  |
| Q1 | ref | ref | ref | ref | ref | ref | ref | ref | ref | ref |
| Q2 | 1.19(0.73,1.95) | 0.48 | 0.79(0.44, 1.44) | 0.43 | 1.13(0.70,1.81) | 0.61 | 0.74(0.46,1.18) | 0.2 | 0.93(0.58,1.49) | 0.77 |
| Q3 | 1.34(0.81,2.22) | 0.25 | 1.31(0.64, 2.67) | 0.46 | 1.45(0.90,2.35) | 0.13 | 1.07(0.67,1.71) | 0.78 | 0.92(0.56,1.51) | 0.74 |
| Q4 | 2.24(1.22,4.11) | **0.01** | 1.63(0.67, 3.95) | 0.28 | 2.14(1.20,3.83) | **0.01** | 1.81(1.13,2.91) | **0.02** | 0.85(0.46,1.59) | 0.61 |
| **TyG-WHtR(continuous)** | 1.92(1.52,2.43) | **<0.0001** | 1.44(1.03, 2.02) | **0.03** | 1.47(1.12,1.91) | **0.01** | 1.69(1.29,2.21) | **<0.001** | 1.16(0.86,1.58) | 0.33 |
| **TyG-WHtR(categorical)** |  |  |  |  |  |  |  |  |  |  |
| Q1 | ref | ref | ref | ref | ref | ref | ref | ref | ref | ref |
| Q2 | 1.31(0.83,2.05) | 0.24 | 0.89(0.50, 1.57) | 0.67 | 1.07(0.69,1.68) | 0.75 | 0.62(0.40,0.97) | 0.04 | 0.86(0.53,1.39) | 0.53 |
| Q3 | 1.71(0.99,2.98) | 0.06 | 1.74(0.88, 3.44) | 0.11 | 1.71(1.05,2.78) | **0.03** | 1.20(0.73,1.96) | 0.47 | 0.80(0.46,1.41) | 0.44 |
| Q4 | 2.72(1.46,5.09) | **0.002** | 1.97(0.81, 4.80) | 0.13 | 2.20(1.30,3.71) | **0.004** | 2.13(1.24,3.64) | **0.01** | 0.78(0.41,1.49) | 0.44 |

Model 1was unadjusted, Model 2 was adjusted for age, gender and race, Model 3 was adjusted for age, gender, race, smoking, drinking, exercise, marriage, education, PIR, SBP, DBP, BMI, LDL, TC, TBIL, SUA, eGFR, and chest pain. Abbreviations: TyG *Triglyceride-glucose index*, TyG-WC *Triglyceride-glucose-waist circumference index,* TyG-WHtR *Triglyceride-glucose-waist-to-height radio index.*


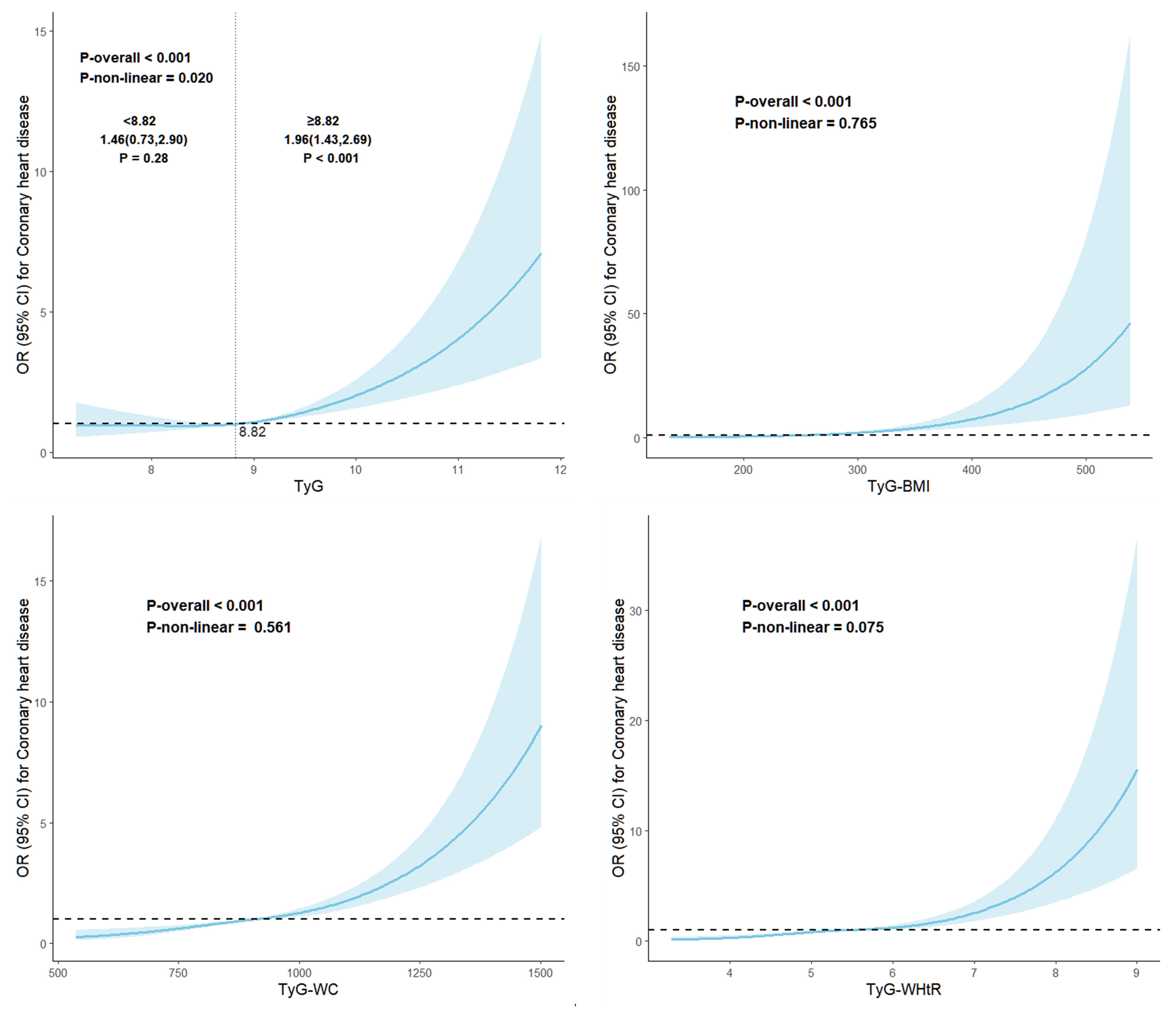


**Supplementary Figure 1** Restricted cubic spline curves for TyG, TyG-BMI, TyG-WC and TyG-WHtR associated with coronary heart disease.


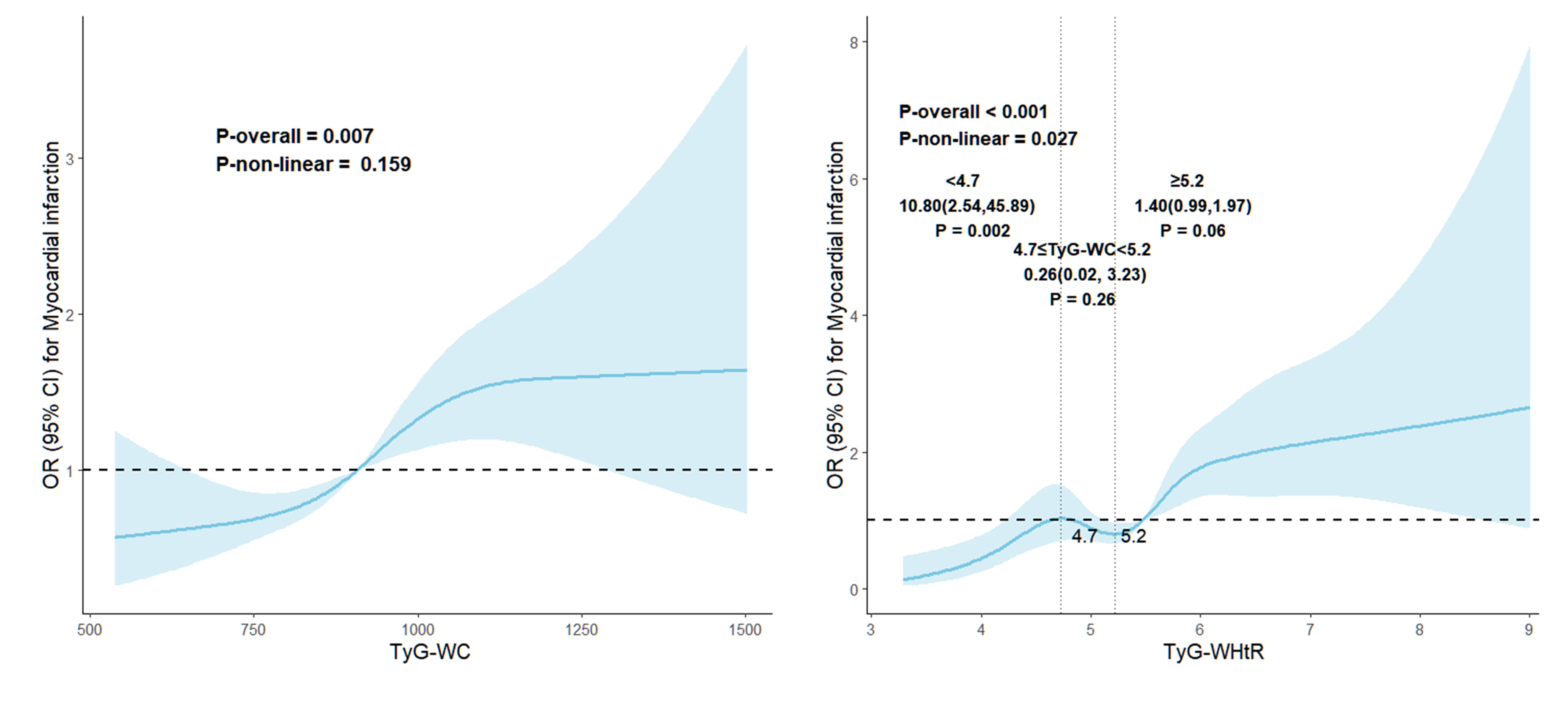


**Supplementary Figure 2** Restricted cubic spline curves for TyG-WC and TyG-WHtR associated with myocardial infarction.


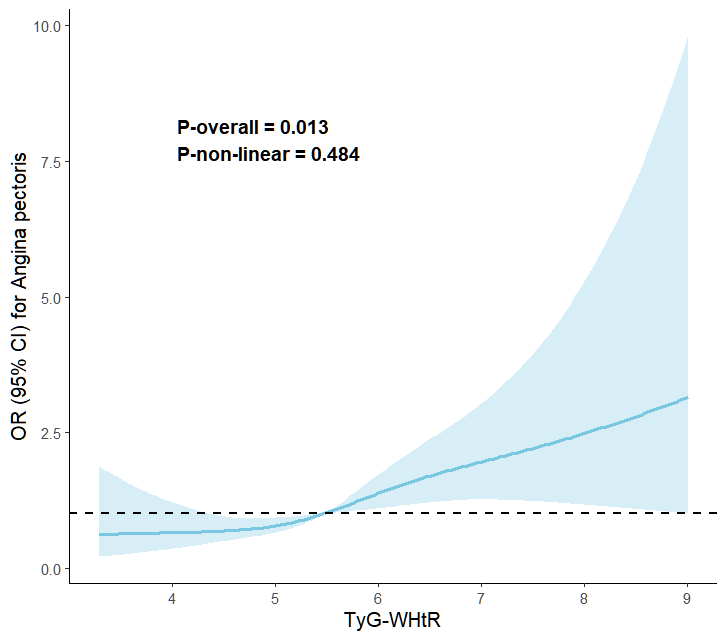


**Supplementary Figure 3** Restricted cubic spline curves for TyG-WHtR associated with angina pectoris.


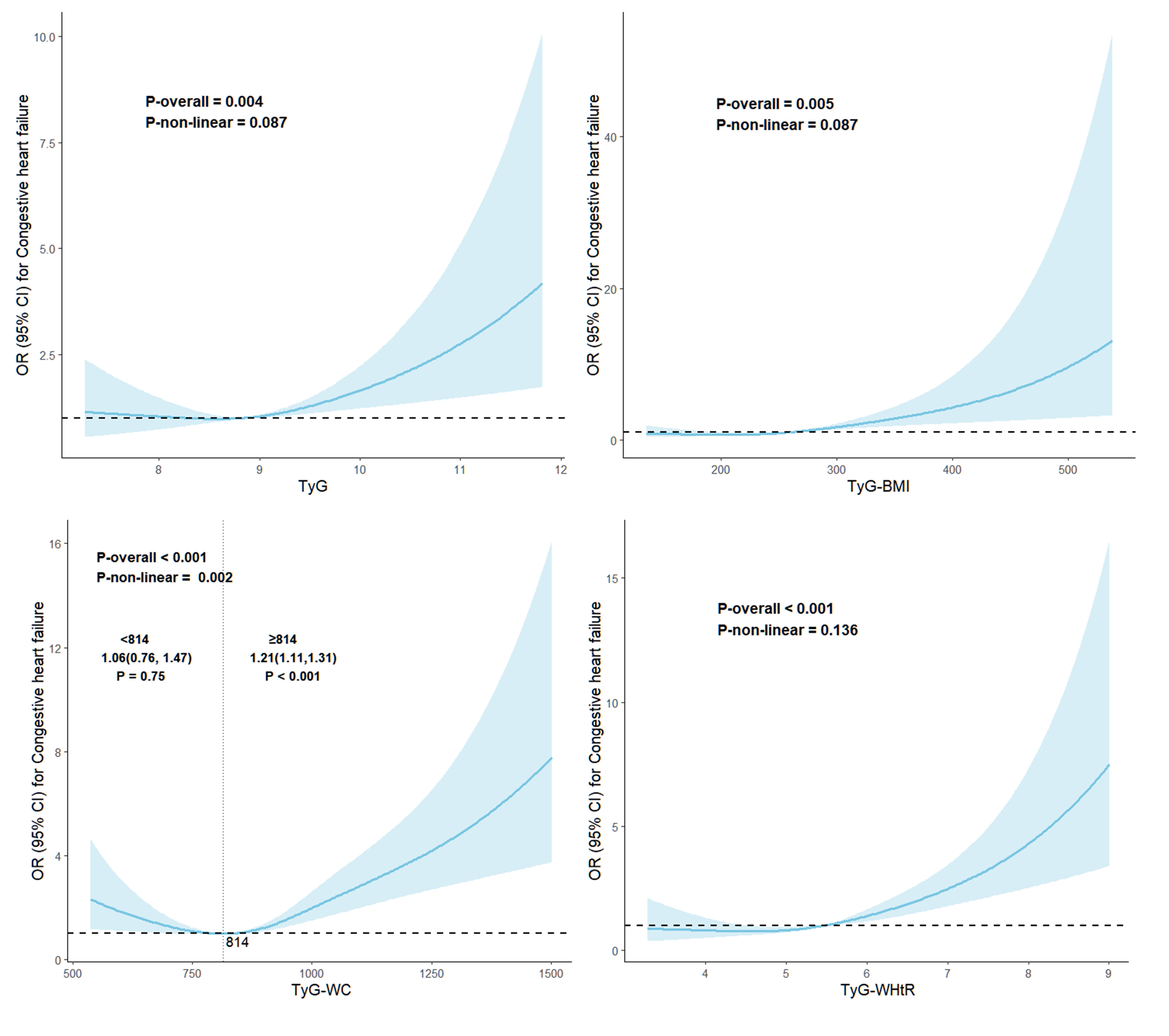


**Supplementary Figure 4** Restricted cubic spline curves for TyG, TyG-BMI, TyG-WC and TyG-WHtR associated with congestive heart failure.

**Supplementary Table 2** Threshold effect analysis of TyG, TyG-WC and TyG WHtR with total-CVD

|  | **Total-CVD** | |
| --- | --- | --- |
| **Inflection point** | **OR (95% CI)** | ***P* value** |
| **TyG** |  |  |
| TyG < 8.82 | 1.25(0.70,2.23) | 0.44 |
| TyG ≥ 8.82 | 1.70(1.29,2.22) | **<0.001** |
| **TyG-WC(continuous, per 50 increase)** |  |  |
| TyG-WC < 790 | 1.46(1.11,1.92) | **0.01** |
| 790 ≤ TyG-WC < 872 | 0.87(0.54,1.39) | 0.55 |
| TyG-WC ≥ 872 | 1.15(1.07,1.24) | **<0.001** |
| **TyG-WHtR** |  |  |
| TyG-WC < 4.7 | 2.53(1.42,4.50) | **0.002** |
| 4.7 ≤ TyG-WC < 5.2 | 0.56(0.05,6.34) | 0.63 |
| TyG-WC ≥ 5.2 | 1.79(1.41,2.27) | **<0.001** |

**Supplementary Table 3** Threshold effect analysis of TyG with coronary heart disease

|  | **Coronary heart disease** | |
| --- | --- | --- |
| **Inflection point** | **OR (95% CI)** | ***P* value** |
| **TyG** |  |  |
| TyG < 8.82 | 1.46(0.73,2.90) | 0.28 |
| TyG ≥ 8.82 | 1.96(1.43,2.69) | **<0.001** |

**Supplementary Table 4** Threshold effect analysis of TyG-WHtR with myocardial infarction

|  | **Myocardial infarction** | |
| --- | --- | --- |
| **Inflection point** | **OR (95% CI)** | ***P* value** |
| **TyG-WHtR** |  |  |
| TyG-WC < 4.7 | 10.80(2.54,45.89) | **0.002** |
| 4.7 ≤ TyG-WC < 5.2 | 0.26(0.02, 3.23) | 0.26 |
| TyG-WC ≥ 5.2 | 1.40(0.99,1.97) | 0.06 |

**Table S5** Threshold effect analysis of TyG-WC and TyG WHtR with congestive heart failure

|  | **Congestive heart failure** | |
| --- | --- | --- |
| **Inflection point** | **OR (95% CI)** | ***P* value** |
| **TyG-WC(continuous, per 50 increase)** |  |  |
| TyG-WC < 814 | 1.06(0.76, 1.47) | 0.75 |
| TyG-WC ≥ 814 | 1.21(1.11,1.31) | **<0.001** |


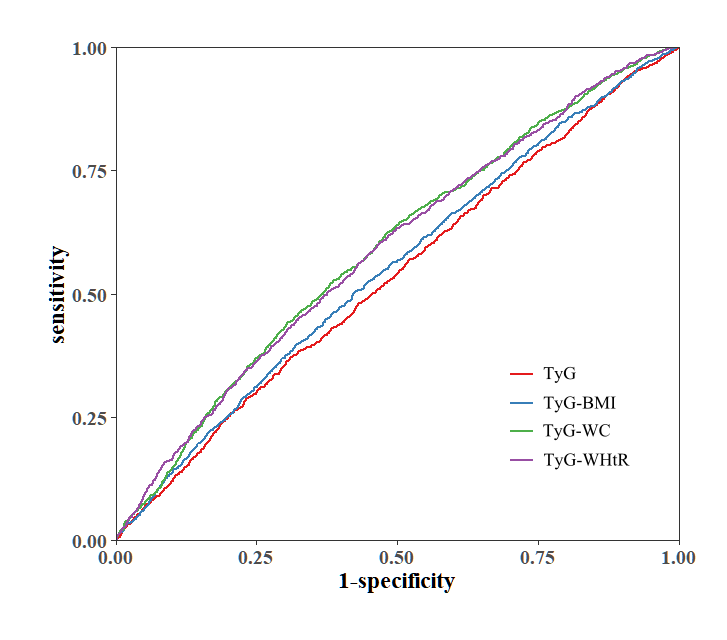


**Supplementary Figure 5** ROC curves of TyG, TyG-BMI, TyG-WC and TyG-WHtR for discriminating total-CVD.
